# Mental Health Nurses' Experiences of Caring for Patients Suffering from Self-Harm

**DOI:** 10.1155/2014/905741

**Published:** 2014-11-13

**Authors:** Randi Tofthagen, Anne-Grethe Talseth, Lisbeth Fagerström

**Affiliations:** ^1^Lovisenberg Diaconal University College, Oslo, Norway; ^2^The Department of Health and Care Sciences, Faculty of Health Sciences, University of Tromsø, The Artic University of Norway, Norway; ^3^Faculty of Health Sciences, Buskerud and Vestfold University College, Norway

## Abstract

The aim of this study was to explore mental health nurses' experiences of caring for inpatients who self-harm during an acute phase. The setting was four psychiatric clinics in Norway. Fifteen mental health nurses (MHNs) were recruited. Semistructured interviews comprised the method for data collection, with content analysis used for data analysis. Two main categories emerged: challenging and collaborative nurse-patient relationship and promoting well-being through nursing interventions. The underlying meaning of the main categories was interpreted and formulated as a latent theme: promoting person-centered care to patients suffering from self-harm. How MHNs promote care for self-harm patients can be described as a person-centered nursing process. MHNs, through the creation of a collaborative nurse-patient relationship, reflect upon nursing interventions and seek to understand each unique patient. The implication for clinical practice is that MHNs are in a position where they can promote patients' recovery processes, by offering patients alternative activities and by working in partnership with patients to promote their individual strengths and life knowledge. MHNs strive to help patients find new ways of living with their problems. The actual study highlighted that MHNs use different methods and strategies when promoting the well-being of self-harm patients.

## 1. Introduction

Self-harm is an expanding health problem and is in many cases a hidden behavior. Direct self-harm has been described with different terms, including deliberate self-harm, self-injury, attempted suicide, self-mutilation, or parasuicide [1, 2]. It is difficult to measure accurately how many people self-harm. In a case study in Norway with 4060 students aged 15-16, 10.3% of girls and 3.1 of boys engaged in self-harm [3]. In the UK it is estimated that self-harm affects one out of every fifteen adolescents [4]. Self-harm among south Asian women in the UK is higher than among the white population [5], yet according to one recent study white adolescent females seem to be overrepresented in many self-harm studies [2].

Direct self-harm can encompass many different forms such as cutting, burning, self-hitting, strangulation, hair pulling, aggravation of chronic wounds, and/or insertion of objects into the body. In a recent study of acute inpatients, the most common form of self-harm involved breaking the skin. Women are more likely to use methods of restricting their breathing, whereas men are more likely to use outwardly aggressive methods [6]. The more individuals injure themselves, the more likely they will become addicted to self-harm [7]. Self-harm can thus be described as a long-term illness and, consequently, many people suffering from self-harm must learn how to cope with their illness. Self-harm is a serious health problem in mental health care [8] and may also be connected to a clear suicidal risk [9–11]. Nonetheless, James et al. [6] find that of the self-harm episodes that adult inpatients in psychiatric care engage in, women with no suicidal risk comprise the largest group. The risk for repetitive self-harm and suicide is greatest the first two years after a first episode of self-harm and this risk may persist over a period of several years [12].

A systematic database search in Cinahl, (Ovid) Medline and PsykLit, using the keywords self-harm/self-injury/self-mutilation, acute mental health care/psychiatric ward, inpatient, and mental health nurse/psychiatric nurse, reveals that only a few studies have focused on nursing interventions for inpatients suffering from self-harm. Earlier studies show that professionals experience caring for patients engaging in self-harm to be challenging, with regard to the nurse-patient relationship [13–18]. Wheatley and Austin-Payne [19] find that unqualified nursing staff report more negativity and worry in working with patients who display self-harm than qualified staff. It seems that better nursing attitudes toward self-harm can be promoted through nursing education and supervision [20, 21]. A shift in nursing attitudes toward self-harm is underway. Nurses no longer expect patients to totally cease self-harm behaviors but instead adopt a collaborative approach with their patients to reduce the frequency or severity of harm [22] and establish a team to support patients in engaging in safe self-injury [23]. According to Holley et al. [23], the implementation of a supporting approach has reduced nurses' anxiety when caring for self-harm patients.

The traditional way of caring for self-harm inpatients in acute mental health care is through restrictive actions [4], such as the removal of knives, razor blades, belts, shoelaces, or lighters, in combination with systematic and rigorous observation [2]. Another study describes [6] that the most common interventions for self-harm are verbal deescalation and manual restraint for inpatients in psychiatric wards. Controlling patients' self-harm over time through seclusion or restriction appears to be ineffective and counter-therapeutic and breaks down the therapeutic relationship [4].

Different approaches have been introduced where patients are taught alternative actions or are allowed to self-harm in the clinical setting [4, 24, 25] instead of controlling or using force with self-harm patients [1, 26]. Stewart et al. [27] find that an association between constant observation and reduced self-harm does not exist. Thus one alternative method is flexible observations. This method is a shift away from the routine use of constant monitoring and involves patients and nursing staff in active and creative problem solving and skills acquisition and may reduce the risk of “ill will” arising between patients and staff [28].

The experienced attitude and relationship of nurses to individuals in their care who self-harm is a major professional and ethical issue in nursing, and antipathy for the self-harming person is a barrier to improving care. It is important that nurses maintain an empathetic presence and guide patients through their self-harm and accept, support, and have confidence in the patients as individuals [4]. Improving patients' self-esteem will in the long-term reduce distress and develop restorative coping acts as an alternative to repetitive self-harm. Coping is not merely a way to respond to a provoking event but also dictates the associated emotion, making the recognition of effective alternative patterns of action all the more important [29].

In the facts that self-harm is an increasing health problem and that changes in care approaches for self-harm patients are occurring, a qualitative study of mental health nurses' (MHNs) experiences of caring for such patients is motivated. Our assumption is that valuable knowledge about caring for self-harm patients is embedded in MHNs' experiences. The aim of this study was therefore to explore and describe MHNs' experiences of caring for inpatients who self-harm during an acute phase.

## 2. Theoretical Background

A person-centered approach is a valuable perspective for nursing [30]. In a person-centered approach, the patient is placed at the center of all care; the patient's experience of health and ill-health is taken into consideration during care and treatment and a focus is placed on an active collaboration between the nurse and patient [31]. The patient needs to be understood as a person [31]. Different models exist for implementing person-centered care across multiple health care settings [32]. Originally introduced into acute mental health nursing, the Tidal model [31, 33, 34] is now recognized as a mid-range nursing theory [31]. In the Tidal model a range of focused assessments generate person-centered interventions that emphasize a person's extant resources and capacity for solution-finding [34]. An open, honest, nonjudgmental, and supportive therapeutic alliance promotes personal development, from fragmentation into wholeness and from despair into hope [31]. Barker and Buchanan-Barker [35] prefer to conceptualize recovery as a process of assisting people to recover their personal identity through telling their own story in their own voice. The challenge for nurses is to facilitate healing through carefully understanding and creating learning out of patients' experiences.

## 3. Materials and Methods

### 3.1. Design

This study has a qualitative exploratory and descriptive design.

### 3.2. Study Setting and Participants

The study setting consisted of five adult acute care units at four psychiatric clinics in Norway. Norway does not currently have any national guidelines for the treatment and care of self-harm patients, and for this reason various clinics were included in this study to gain a broader-access, representative, and comprehensive data material of MHNs' experiences.

Purposive sampling was used. Inclusion criteria were that participants were employed 100% at an acute inpatient psychiatric unit for more than three years as a nurse specialized in mental health nursing (MHN; three-year Bachelor's degree plus one-year specialization training) or as a registered nurse (RN) with extensive work experience in caring for self-harm patients. Nurse leaders and specialist nurses with responsibility for development and quality assurance were excluded.

The top managers at the four psychiatric clinics were given written information about the project and gave the permission for the study. Each nurse manager at the five units recruited participants who met the* inclusion criteria*, and information sheets were used to inform participants about the study. At the beginning of each interview Randi Tofthagen, who performed all interviews, gave participants additional information, including information regarding ethical principle. A total of 15 participants, 2 men and 13 women, gave their written informed consent to participate in the study. Of these, 12 were MHNs and three were RNs with extensive work experience, included due to the small number of MHNs. The participants had worked in acute psychiatric care between 1 year and 14 years (mean 5.1 years). For the purposes of this study, we hereafter only use the term MHN.

### 3.3. Data Collection

The method for data collection was semistructured interviews, conducted during autumn 2010 and the spring of 2011. The semistructured interviews lasted from 45 to 90 minutes. The interview started with an open question regarding the participant's experiences of caring for self-harm patients. The interviews were conducted as a dialogue, and the order of topics was tailored to each individual participant. An interview guide was used during the interviews and the following themes were included in the dialogue between the participant and Randi Tofthagen: MHNs' experiences of caring for self-harm patients and the patients' expressions of self-harm, the use of force, what inhibits or promotes self-harm patients' coping abilities in relation to self-harm, and how self-harm affects MHNs' own feelings. Individual follow-up questions were asked during the interviews to expand and deepen the participants' spontaneous answers to the interview guide themes. The interviews were audio-recorded and transcribed verbatim into text (120 pages) by Randi Tofthagen.

### 3.4. Data Analysis

The method for data analysis was manifest content analysis, followed by latent content analysis inspired by Graneheim and Lundman [36, 37]. The inductive manifest content analyses process in the actual study can be described as a condensation-abstraction process consisting of seven steps. When using inductive content analysis, categories are created from raw data without a theory-based categorization [38]. In the first step, the researchers read and re-read the transcribed interview material to obtain an overall understanding of the content of the interviews, that is, participants' experiences of caring for self-harm inpatients. In the second step the focus lays on the text, which was divided into meaning units. A meaning unit can be one or several words, sentences, or paragraphs that are related to one another through aspects relevant to the aim of the study with regard to content or context [36, 37] (Table 1). In step three, the meaning units were condensed, and in the fourth step the condensed meaning units were compared, discussed, and labeled with codes. In the fifth step, the codes were abstracted, compared, and sorted into subcategories. Ten categories were created from the similarities and differences seen between the subcategories in step six. In step seven, ten categories were formulated into two main categories: challenging and collaborative nurse-patient relationship and promoting well-being thorough nursing interventions (Table 2). In the last phase of the analyzing process, the latent theme was created from a deeper interpretation of the underlying meaning of the subcategories and categories and formulated as follows: promoting person-centered nursing to inpatients suffering from self-harm.

## 4. Results

The results of the manifest content analysis can be described with two main categories: (1) challenging and collaborative nurse-patient relationship and (2) promoting well-being through nursing interventions (see Table 2).

### 4.1. Challenging and Collaborative Nurse-Patient Relationship

The first main category contained four subcategories: caring attitude toward the patient, bearing hope for recovery, being in a reflective dialogue to promote the patient's verbal expressions, and being emotionally affected by self-harm patients.

#### 4.1.1. Caring Attitude toward the Patient

The participants wanted to understand and see the person behind “the suffering human being” who has been mentally harmed previously in life and who because of this is vulnerable in relationships with other people. In order to be able to help the patient and ensure that patient follow-up does not become incidental it is necessary to understand and know the patient as a person:

*“Understand why this patient self-harms - who you have in front of you (i5).”*

A caring attitude was expressed; the participants reflected on how the patient is doing and worried when a patient was out on leave or had been discharged. Patients can be ambivalent to whether they live or die, so the harm they cause themselves can fluctuate between self-harm and attempted suicide. The participants described that the patient's self-harm can be mild, moderate, or severe in degree and can lead to death: also that there was a delicate line between life and death. Patients can miscalculate the severity of their self-harm and harm themselves more than intended. The participants experienced that they continuously strive to understand each patient and that patients can change their self-harm patterns, with the end result that the participants were not always able to recognize patients' new patterns and/or methods:

*“But there is, to be sure, a seriousness in it…at the same time that it can, to be sure, change. Some do not need to be so serious and suddenly it is something else then (i5).”*

Not appearing to be judgmental or guild inducing in the nurse-patient relationship occupied the participants. In the fact that the participants experienced that patients often feel ashamed of their self-harm, the participants sought to circumvent patients' low self-esteem and instead promote trust and a dialogue:

*“How well you know the patient and they feel safe with you - then we can, in a way, put some more words into it - I think (i8).”*

#### 4.1.2. Bearing Hope for Recovery

The participants stated that it is important to be attentive to patients' experiences, see the individual, and believe in the patient's capabilities and rational sides. They sought to be a friendly and respectful presence and convey hope and also maintained the belief that patients can improve. The participants sought to carry the projections they feel in the relationship,to persevere and withstand the relationship and bear hope regarding the patient's recovery when the patient him/herself is unable to envision such occurring:

*“That we give a vision of something…it can actually also work…but I have completely accepted that you are not thinking of it now (i15).”*

It can be time-consuming to inspire hope in others, according to one participant. When a patient is suffering from physical unease and unable to verbalize why he/she engages in self-harm, it is difficult for him/her to understand that his/her need to self-harm can be reduced or stopped. According to the participant, this entails being a cotraveler during repeat hospital/care admissions and guiding the patient's recovery as a process or a learning situation for the patient:

*“It is difficult to converse when they do not know anything else than anger… a lump in the stomach or something like that. But what I can also say here is that there is help available. Try to create hope that there can be improvement in the future. But it can take time then (i12).”*

#### 4.1.3. Being in a Reflective Dialogue to Promote the Patient's Verbal Expressions

The participants spent time with each patient and engaged the patient in a dialogue about the patient's situation: what signs appear when a patient feels the urge to self-harm, and whether the patient should communicate with nurses before or after an act of self-harm. They were clearly focused on having a reflective dialogue that could promote the patients' verbal expressions.

The participants experienced that a patient can learn to manage his/her emotional fluctuations in other ways than through self-harm. They sought to help patients verbalize those feelings related to the self-harm situation. Patients are vulnerable and the participants demonstrated sensitivity to patients' various forms of communication. As a consequence, they therefore sought to help patients articulate their feelings through dialogue instead of self-harm:

*“It is a type of helplessness in the relationship. The feelings…you cannot express yourself. You are not capable of sorting and everything is chaos. They need help putting it into words (i8).”*

Patients are vulnerable and watchful for transgressions, so it is therefore important that MHNs do not reject patients but instead demonstrate sensitivity toward patients' forms of communication. The participants indicated that they sought an alliance with the patient and sought to understand the patient and his/her self-harming behavior.

#### 4.1.4. Being Emotionally Affected by Self-Harm Patients

The participants experienced being in a relationship with a self-harm patient as being unpleasant, provocative, and/or challenging at times. They described that patient relapses could be experienced as a “defeat.” Thus MHNs can become discouraged or experience a sense of powerlessness. Furthermore, the participants even expressed that the patient's emotions can be projected (transferred) onto MHNs themselves, and that MHNs' negative feelings can incite self-harm in a patient. The participants also experienced that patients can crave a personal, private closeness that MHNs are unable to provide:

*“My feelings can be chaotic of lot of what I am thinking can be projections from the patient. Well, I am able to separate myself more now than when I was new for example, capable of separating myself from the patient's feelings, for example, to stop this projection storm (i12).”*

According to participants, after caring for a patient and putting effort into helping the patient recover, the patient's relapses can be experienced as a “defeat” and can give rise to a feeling of inadequacy with regard to promoting recovery. Instead of engaging patients in a close relationship, the participants attempted to create a boundary between themselves and the patients (but without rejecting the patients), so that it was possible to separate themselves from their patients and promote a professional relationship.

The participants mentioned experiencing a sense of intuition at times, a “gut feeling” in regard to a patient's impending self-harm. They described this kind of intuition as a combination of feelings, experience, and knowledge through which they actively explore the here-and-now of a situation. Knowledge of various psychological defense mechanisms and countertransference and the ability to distinguish feelings and emotions are examples of the knowledge that the participants used during the reflection process:

*“Gut feeling comes very fast - a combination of knowledge, and experience in a way. You speak on and off about that you “smell” things then. You can, to be sure, capture [it] (i4).”*

### 4.2. Promoting Well-Being through Nursing Interventions

The second main category includes six subcategories: evaluating and following-up of triggers, observing signs of risk for self-harm, searching for prevention activities, allowing and/or preventing external self-harm, taking responsibility for patients' wounds and injuries, and evaluating need for medication.

#### 4.2.1. Evaluating and Following-Up of Triggers

Triggers can include situations, thoughts, and/or feelings that can generate a need for direct self-harm: for example, being alone, rejection, conversations with a physician or nurse, evenings, and/or private circumstances. The participants sought to discover what triggers a patient's self-harm and experienced that they can also trigger a patient's self-harm by not being present or through a lack of understanding for the patient's situation and behavior:

*“You are perhaps occupied for [15 minutes] with another patient. They then go and hide themselves and self-harm. It could be, to be sure, that it is so difficult when there is no one there who distracts that they must just do it - that they cannot hold out (i1).”*

External control of patients' behavior can trigger self-harm. This can lead to a struggle between the patient and MHNs for control:

*“Know of one who carried on tremendously who was blue and - yes, the more we went in and used control and force the more she banged her head and it became very difficult (i8).”*

#### 4.2.2. Observing Signs of Risk for Self-Harm

The participants described how they are able to notice whether an act of self-harm is about to happen, is happening, or has occurred. It is therefore important that MHNs continuously observe and identify warning signs: for example, when patients, while attempting to conceal their actions, withdraw from social interaction or engage in restless walking, picking or scratching the body:

*“Perhaps a glance when I go by - a little elusive - see me now. Perhaps I can get a feeling of - and then they retreat, are gone a long time. Think that now it is a long time since I have seen her so then I must go and [have a] look (i1).”*

#### 4.2.3. Searching for Prevention Activities

When triggers or warning signs were observed, the participants attempted to avert patients' self-harm through activities. Activities could include going for a walk, play, watching TV, conversations, boxing, crafts, billiards, squeezing objects, and playing the drums. Active diversion is an expression of care and creates a distance between patients and their suffering and simultaneously teaches patients alternative strategies to self-harm.

To help patients cope, the participants worked with the patients, encouraged them to articulate their need for self-harm, and responded positively when patients mastered such strategies:

*“Speak with the patients about what can help. Is it to take a walk, write it down, is it knitting, is it quickly back and forth in the corridor, sedatives - so, what is it that can help? (i5).”*

Still, according to the participants many patients are unaware of what triggers their self-harm. They do not have a verbal language with which to express their pain and instead communicate with their bodies. Patients can also have difficulty speaking out when the need to self-harm starts to develop. For example:

*“A girl started with it when she was extremely young - almost before she could speak (i3).”*

#### 4.2.4. Allowing and/or Preventing External Self-Harm

Patients are searched when being admitted to a unit, and the participants sought to ensure that this occurred in partnership with the patient and in a voluntary spirit. Patients are only allowed a limited number of personal belongings. Staff removed and stored lighters, sharp objects, narcotics, and more. The participants experienced that diverse attitudes toward whether or how much a patient is allowed to self-harm while in care varied between units and even MHNs. The study results indicate that two different approaches to setting boundaries for patients' actions exist.

The first approach is that patients are not allowed to self-harm at all. All self-harm is stopped through the use of control, such as physically holding patients, medication, close monitoring, the use of metal detectors, seclusion, or restraints:

*“A patient who is restrained who is engaging in self-harm does not, to be sure, always receive so much medication, but - that the patient should have it better and be calmer (i5).”*

The second approach is that patients are allowed to harm themselves to a mild or moderate degree while admitted to a unit; patients often possess or can access tools with which to harm themselves and are not physically restrained. The participants experienced that allowing patients to harm themselves to a mild or moderate degree can prevent serious self-harm in that it relieves patients' inner mental pain. Some participants pointed out that this way of relating to self-harm can help patients master and cope with their problem. The participants stated that some variation between units exists; on some units patients were not allowed to self-harm in the common areas of the unit. In general, patients were encouraged to relinquish their tools once the self-harm act was completed:

*“We do not stop it because for some it is important to see blood (i9)”;*

*“Simply with the [physical environment] that there are locked doors and they are not allowed to have many different things so, it is difficult to give them the responsibility they should have to learn and cope with this. How they should learn to master to not cut themselves when they do not have anything to cut themselves with (i1).”*

The participants reported that serious self-harm that can lead to suicide is always stopped, whether through the use of seclusion or restraints or constant observation for shorter and longer periods of time.

#### 4.2.5. Taking Responsibility for Patients' Wounds and Injuries

Care and treatment was provided in accordance with the degree of severity of wounds and injuries. Mild injuries were dressed with adhesive bandages, whereas the most severe injuries could require the suturing of larger areas of skin. This created a sense of “calm” in regard to the wound/injury, with a focus on the prevention of infection. The participants experienced that wound care can be considered a form of communication, yet disagreed as to whether they should call attention to the wound/injury or not:

*“I speak with the patient but I do not go into what triggered it. I do not want the patient to start to resist - the wound care. I want to be finished with it. It is not so easy to know what can provoke it (i2).”*

#### 4.2.6. Evaluating Need for Medication

Together with a physician the participants assessed whether or not medication could help patients reduce their mental pain. The participants described that psychosis could be easier to treat than emotionally unstable personality disorders, since neuroleptic medication reduces psychotic symptoms that appear to promote the need for self-harm. At times, participants used medication to either “protect” patients from self-harm or “knock out” patients so that they became calm or sleepy:

*“Sometimes it can help to completely knock out the patient [with medication]. The patient is given Atrovan or a powerful antipsychotic medicine (i7).”*

The participants indicated that medicating patients can be a way to alleviate patients' expressions of pain when uncertainty exists as to what interventions should be implemented:

*“I think you can be a little too easy with this, actually that you too quickly give medication when we notice that we are unsure (i7).”*

### 4.3. The Latent Theme

In the last phase of the analysis process, the meaningful content of the subcategories and categories was interpreted. The participants experienced that they guide many patients to well-being and reduced rates of self-harm, despite disappointing relapses and challenging feelings. The nursing process started with seeking to understand the self-harm patient, who often balances between life and death, and how each unique patient's well-being can be promoted through the creation of a collaborative nurse-patient relationship and through a focus on person-centered nursing interventions that promote the patient's recovery process. The underlying meaning of the participants' experiences of helping and caring for direct self-harm patients was interpreted and then formulated as a latent theme: promoting person-centered nursing to inpatients suffering from self-harm.

## 5. Discussion

In the actual study, participants described self-harm as a fluid phenomenon that changes in intensity and character, from mild self-harm to obvious suicide attempts. The participants sought to understand and confirm the person behind “the suffering human being” who has been mentally harmed previously in life and who because of this is vulnerable in relationships with other people.

This study shows that MHNs seek person-centered solutions and ways of promoting individual patients' well-being. Health in person-centered nursing is linked to personal well-being, and standard nursing interventions may not be appropriate for every nursing interaction in person-centered nursing [30]. According to Barker and Buchanan-Barker [31], the Tidal model starts with an evaluation of how disturbed a person is, his/her need for security, and whether the person can begin to tell his/her story. Interventions through acute services may limit the risk of physical harm but often fail to address the patient's underlying emotional insecurity [31]. Researchers have studied the Tidal model in various acute mental health care studies, and the results from these studies indicate that a reduction in self-harm and suicide attempts occurs when the Tidal model is employed [40, 41]. In a study by Ruddick [42], MHNs sought to help patients explore their inner world so that fragmentation could be turned into wholeness, despair into hope, and conflict into harmony.

An interesting finding from this study is that the participants consciously observed and focused on interpreting and understanding the triggers and signs of self-harm and therefore had good possibilities to prevent self-harm in a goal-oriented manner through diversion. The participants sought to promote person-centered nursing by helping patients learn to articulate their pain, recognize their triggers, and/or use other strategies to divert physical suffering and modify their behavior. The participants also sought to help develop patients' ability to cope with internal and external triggers, which can reduce their need for self-harm. The participants promoted an individual learning process, which included an attempt to truly understand and support each individual patient, and showed patients various types of self-treatment in order to empower the patients to engage in less direct self-harm over time.

A central finding is the varying approach to whether patients should be allowed to engage in mild or moderate self-harm when admitted to a unit. At two clinics, patients were allowed self-harm tools but were encouraged to return them to nurses when not in use. The participants wanted to be present with patients both when self-harm was being stopped or being committed. Those participants who allowed patients to self-harm while admitted to a unit often saw that the patients after a short while felt better and experienced self-control over their suffering. Still, staff control can create a power struggle between patients and nurses and, in certain cases, can even cause more self-harm. In this study, participants reported that patients with a history of self-harm received both emotional and practical support and were allowed to self-harm in order to create physical pain and reduce mental distress. Patients accustomed to using knives or razorblades could be given the possibility to continue using these tools. Thus the participants supported the patients in becoming aware of their own feelings [43]. This way of working entails that MHNs must be able to stand the insecurity of transferring some control to the patient. Nevertheless, many people are forcibly admitted to acute mental health units because they are a danger to themselves [44]. Even though patients are undergoing an acute crisis, the danger for suicide attempts or self-harm is still very high six months after a self-harm episode [45], and it is important to prevent suicide. Many patients bring equilibrium into their lives and achieve peace through self-harm, but because of physiological dependency the need for physical pain increases. Simeon et al. [46] found that cutting can create a dream-like condition for an individual, which can be compared to illicit drug use, and that this can promote repetitive self-harm because of the need to experience this mood change. Consequently, a systematic evaluation of suicide risk should be included in normal treatment routines.

A nonjudgmental approach by MHNs emerged as being central to the creation of a dialogue with patients where patients feel accepted. It can be inhibitory if MHNs do not know the patient they are caring for and follow-up care can become more provisional. It is also important for MHNs to have a professional role and balance closeness and distance in nurse-patient relationships, which should be predictable and supportive over time. This way of relating to patients promotes participation and empowerment. A partnership, as part of a therapeutic relationship, where mutual trust, humanistic caring, and a nonjudgmental attitude exist, promotes person-centered nursing [47, 48].

The participants believed in patients' abilities and rational sides and bore hope for their recovery, even when the patients themselves did not have such hope. Lindgren et al. [49] stressed the importance of hopefulness and that patients feel confirmed and receive support as determinants in the reduction and discontinuation of self-harm. Weber [50] found that patients have hope in the construction of their identity, something that supports that MHNs should use hope as an intervention. By showing hope in relation to patients, trust and faith are demonstrated in patients and patients' own resources.

Person-centered mental health nursing is more than the therapeutic relationship; it also includes the way services and organizations work [51]. It is problematic that most units do not allocate time for follow-up. Preventing repetitive self-harm requires follow-up from all members of the health care team over time and, primarily, from the MHN who cared for the self-harm patient during the patient's acute phase [52].

The participants in this study described the nurse-patient relationship as being very challenging. Through their manner, patients stimulate feelings in MHNs that can awaken powerful extremes: from compliance to chaos. The actual study also shows the importance of the care philosophy of the nursing community as a whole and even nurses' ability to cooperate among themselves so that patients do not perceive any staff disagreement, which can promote patient anxiety. Earlier studies have shown that the greatest stress factor to affect MHNs is their feelings of responsibility for patients' self-harm [53]. In this study the participants facilitated a reflective dialogue with their patients and experienced that, over time, their own feelings toward patients were transformed into greater compassion through the relationship. Patients must be allowed to feel difficult emotions and express emotions without being judged in order to develop positive self-esteem and get in contact with themselves [54].

### 5.1. Methodological Considerations

The results of this study are based on a comprehensive and rich material from interviews with fifteen MHNs working at five acute mental health units who, after many years of experience, still had faith in the ability of patients to move toward a feeling of well-being. Data saturation was seen after fifteen interviews when the data repeated. The trustworthiness of the results has been secured through the documentation of the analysis process, where the results are substantiated with data [36, 55]. The researchers, all MHNs with clinical and research experience, discussed the steps of the condensation-abstraction process. All three researchers examined the data independently and agreement was obtained regarding the identified main categories and latent theme. In the study, rigor is demonstrated by the inclusion of tables (Tables 1 and 2) that illustrate the condensation-abstraction process of how the main categories were developed from the raw data to promote sensitivity in the abstraction, which is a form of validation [37]. Content analyses served the purpose of the study. However, a more hermeneutic method might have highlighted other aspects of the study.

## 6. Conclusions

MHNs seek to understand the self-harm patient, who often balances between life and death. Each patient's unique well-being can be promoted by creating a collaborative nurse-patient relationship and person-centered nursing interventions. We found that self-harm can change in character and intensity and that caring for patients who are suffering from self-harm requires MHNs with advanced clinical competence.

Further research should focus on how MHNs can promote well-being through concrete person-centered nursing interventions, such as wound care, medication, or individual nursing and mastery plans, especially from the patients' perspective. Also, the use of systematic observations and assessments, observance of triggers, and use of diversion while engaged in a reflective dialogue is recommended.

## Figures and Tables

**Table 1:** Examples of the condensation-abstraction process from meaning units to category and main category: challenging and collaborative nurse-patient relationship.

Meaning unit	Condensed meaning unit	Code	Subcategory	Category
*Example 1*				
It is perhaps about that I worry…that I speak with…a person quite simply… Not such a huge injury either… [3]	Speak with the person, not “the self-harmer” and worry	To worry about the person	Worry for the person	Caring attitude toward the patient
*Example 2*				
That the patient receives care in other ways than…when he self-harms [8]	Receive care in another way than with self-harm	To demonstrate care in another way	Care for the patient	

**Table 2:** Mental health nurses' experiences of caring for inpatients suffering from self-harm.

The latent theme	
Promoting person-centered nursing to inpatients suffering from self-harm	
*Main category 1* Challenging and collaborative nurse-patient relationship	*Main category 2* Promoting well-being through nursing interventions

*Categories* Caring attitude toward the patient Bearing hope for recovery Being in a reflective dialogue to promote the patient's verbal expressions Being emotionally affected by self-harm patients	*Categories* Evaluating and following-up of triggers Observing signs of risk for self-harm Searching for prevention activities Allowing and/or preventing external self-harm Taking responsibility for patients' wounds and injuries Evaluating need for medication
